# Chemogenomic Profiling of the Fungal Pathogen Candida albicans

**DOI:** 10.1128/AAC.02365-17

**Published:** 2018-01-25

**Authors:** Yaolin Chen, Jaideep Mallick, Alaa Maqnas, Yuan Sun, Baharul I. Choudhury, Pierre Côte, Lan Yan, Ting-jun-hong Ni, Yan Li, Dazhi Zhang, Roberto Rodríguez-Ortiz, Quan-zhen Lv, Yuan-ying Jiang, Malcolm Whiteway

**Affiliations:** a Biology Department, Concordia University, Montreal, Canada; b Center for New Drug Research, School of Pharmacy, Second Military Medical University, Shanghai, People's Republic of China; c Department of Organic Chemistry, School of Pharmacy, Second Military Medical University, Shanghai, People's Republic of China; d CONACYT, Institute of Neurobiology, UNAM, Juriquilla Campus, Querétaro, Mexico

**Keywords:** Candida albicans, drug interactions, genome analysis

## Abstract

There is currently a small number of classes of antifungal drugs, and these drugs are known to target a very limited set of cellular functions. We derived a set of approximately 900 nonessential, transactivator-defective disruption strains from the tetracycline-regulated GRACE collection of strains of the fungal pathogen Candida albicans. This strain set was screened against classic antifungal drugs to identify gene inactivations that conferred either enhanced sensitivity or increased resistance to the compounds. We examined two azoles, fluconazole and posaconazole; two echinocandins, caspofungin and anidulafungin; and a polyene, amphotericin B. Overall, the chemogenomic profiles within drug classes were highly similar, but there was little overlap between classes, suggesting that the different drug classes interacted with discrete networks of genes in C. albicans. We also tested two pyridine amides, designated GPI-LY7 and GPI-C107; these drugs gave very similar profiles that were distinct from those of the echinocandins, azoles, or polyenes, supporting the idea that they target a distinct cellular function. Intriguingly, in cases where these gene sets can be compared to genetic disruptions conferring drug sensitivity in other fungi, we find very little correspondence in genes. Thus, even though the drug targets are the same in the different species, the specific genetic profiles that can lead to drug sensitivity are distinct. This implies that chemogenomic screens of one organism may be poorly predictive of the profiles found in other organisms and that drug sensitivity and resistance profiles can differ significantly among organisms even when the apparent target of the drug is the same.

## INTRODUCTION

The interaction between microbes and antimicrobial compounds is of considerable interest for both practical and scientific reasons. The discovery and exploitation of penicillin ushered in the era of well-defined antibacterial molecules (1), and drugs against other human pathogens followed from the initial successes of such antibiotics (2). This has had a revolutionary impact on human health. More recently, the rise of antibiotic resistance among a wide variety of human pathogens has put our medical exploitation of antimicrobial compounds at risk, increasing the interest both in drugs with new modes of action and in defining the mechanisms of resistance to find strategies to circumvent such mechanisms (3).

Eukaryotic pathogens have provided particularly challenging targets for antimicrobial compound development, because the underlying molecular processes controlling growth and proliferation of these pathogens are generally conserved with those of the equivalently eukaryotic human host. Thus, compounds that target central cellular functions in eukaryotic pathogens run the risk of targeting host cells as well, with the potential for serious side effects. Compounds that are clinically successful against eukaryotic pathogens have had to exploit unique features of the pathogens; this has tended to limit the classes of compounds that generate successful drugs to combat these pathogens (4).

A variety of Candida species, primarily Candida albicans but including growing numbers of other relatives, such as C. tropicalis and C. krusei, cause a considerable fraction of human fungal disease (5). They can be particularly damaging to individuals with compromised immune systems, and they make up a major component of nosocomial infections in North American hospitals (6). A variety of antifungal drugs have been developed and marketed, and these fall into 4 major classes: the polyenes, the azoles, the allylamines, and the echinocandins. Each of these classes attacks a component of the pathogenic fungi that is distinct from the human host: the polyenes cause membrane leakage through interaction with ergosterol, a membrane component replaced by cholesterol in humans, the azoles and allylamines block the synthesis of ergosterol at different steps in the pathway, and the echinocandins attack the biosynthesis of the fungal cell wall. Overall, these drugs are quite effective and generally have acceptable levels of side effects relative to their ability to treat disease (4). However, none can be considered ideal. For example, there are growing populations of azole-resistant strains arising through mutations or the result of natural resistance in some species (7), and amphotericin B (AmB), the most widely used polyene, can cause potentially serious side effects (8).

It is therefore of considerable interest to researchers to identify antifungal drugs with new modes of action, to find new antifungal targets, and to investigate strategies for overcoming resistance to current drugs. Chemogenomics, which can be defined as the high-throughput investigation at the genomic level of the interaction of small molecules with cells (9), provides a potential tool for such studies. In concrete terms, chemogenomic investigations often involve establishing the relationships between what are typically small molecules or collections of small molecules and a genomically defined organism. These relationships can include finding genes whose expression is modulated by the treatment of a cell with the compound or compounds (10), identifying inactivated genes that confer sensitivity or resistance to a specific chemical or library of molecules, or establishing which chemicals interact physically or functionally with a biologically defined target (11, 12). The analysis of the interaction between small molecules and genomically defined cells is particularly promising for the ascomycete fungi. These fungi typically have small genomes, and several species have been investigated extensively through the construction of libraries of inactivated genes. Saccharomyces cerevisiae, an ascomycete yeast, is the most thoroughly studied of the fungi. Comprehensive gene disruption libraries have been available since 2002 (13), and many large-scale studies linking these deletion strains to chemicals, including antifungal drugs, have been done (10, 14; for reviews, see references 15 and 16). Schizosaccharomyces pombe disruption collections have also been applied to the analysis of chemical interactions (17), and smaller or more focused disruption collections of other fungi have also been investigated (18, 19).

While the connection of model yeast disruption collections to chemical libraries has provided significant insight into the interactions of various compounds with genetic networks, the interaction of compounds with human-pathogenic fungi is of particular interest. The recent development of the GRACE library, an extensive collection of conditional mutants of C. albicans (20), opened up the potential for direct studies with the pathogen. The application of the genome-wide bar-coded heterozygous collection in fitness assays has already identified the mode of action for a number of antifungal drugs (21–23), and the conditional inactivation of genes can be used to probe entire genetic networks connected to such drugs. However, the requirement of tetracycline treatment to shut off gene expression provides the possibility that observed interactions may not simply be the consequence of the interaction of the drug and the inactivated gene but may be influenced by the presence of tetracycline or a tetracycline analog. This complication has been particularly evident in the case of azoles (24).

We made a derivative library from the GRACE collection to create a collection of nonconditional, nonessential inactivated genes. We used this library to probe for genes whose inactivation leads to either sensitivity or resistance to a set of commercial or candidate antifungal drugs. Drugs of the same class generated similar profiles of genes conferring sensitivity and resistance, while different drug classes had very distinct profiles. Intriguingly, comparisons with other fungi showed that the gene networks conferring sensitivity to a particular drug can show dramatic differences among species even though the drug target is the same.

## RESULTS

### Development of a nonconditional derivative collection from the GRACE library.

The GRACE collection of C. albicans conditionally lethal strains provides a powerful tool for functional genomics analysis of this important fungal pathogen (20). The background strain used for the construction of this collection, CaSS1, was developed for the specific purpose of testing the efficacy of antifungal drugs and is derived from SC5314, a clinically isolated strain; it has since been used by multiple groups to study drug interactions with Candida albicans (25, 26; reviewed in reference 27). In the GRACE collection, the *HIS3* marker replaces one allele of a gene of the diploid C. albicans, and the other allele is under the control of a tetracycline-responsive (TetR) promoter. The transactivator of TetR is part of a *URA3*-containing plasmid, which is integrated at one copy of the *LEU2* locus (20). However, the tetracycline-regulated promoter driving the expression of each conditional construct causes some complications—not all genes are totally shut off by the tetracycline-regulated repression circuit, and experiments involving the library must be done in the continual presence of tetracycline or doxycycline to shut down gene expression. The latter point can complicate analyses of strain-drug relationships if there is an interaction between the chemical under study and tetracycline (24). Because of these concerns, we developed a derivative sublibrary of many of the nonessential genes in the GRACE collection lacking the transcriptional activator cassette. This generated a collection of *ura3*^−^ nonconditional null mutants that can be assayed without using tetracycline to repress the expression of the genes. The derivative GRACE 1.0 library of 887 strains was generated as described in Materials and Methods. We tested the sensitivity and resistance of these disruption strains to a variety of antifungal compounds.

### Echinocandins.

Echinocandins represent an important class of antifungal drugs. These compounds disrupt the cell wall of C. albicans by inhibiting the function of Fks1, the key β-1,3 glucan synthetase of the cells (28). We investigated the sensitivity and resistance profiles of the nonconditional mutant collection in response to two echinocandins: caspofungin and anidulafungin.

**FIG 1:**
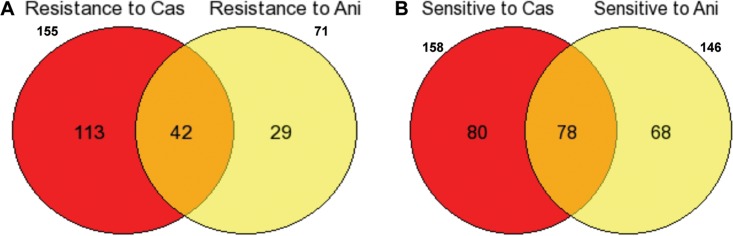
Comparisons of genes showing sensitivity and resistance to caspofungin (Cas) and anidulafungin (Ani). (A) Forty-two strains showed resistance to both caspofungin and anidulafungin. (B) Seventy-eight strains showed sensitivity to both caspofungin and anidulafungin.

Both compounds gave clear sensitivity and resistance profiles, and the overlap in activity between the two compounds was considerable. As shown in Fig. 1A, 149 strains were found to show resistance to caspofungin, 63 strains showed resistance to anidulafungin, and 39 strains showed a common resistance to both compounds. Similarly, the sensitivity profiles of the two compounds had considerable overlap: 158 strains showed sensitivity to caspofungin, 144 strains showed sensitivity to anidulafungin, and 78 strains showed sensitivity in common (Fig. 1B).

**FIG 2:**
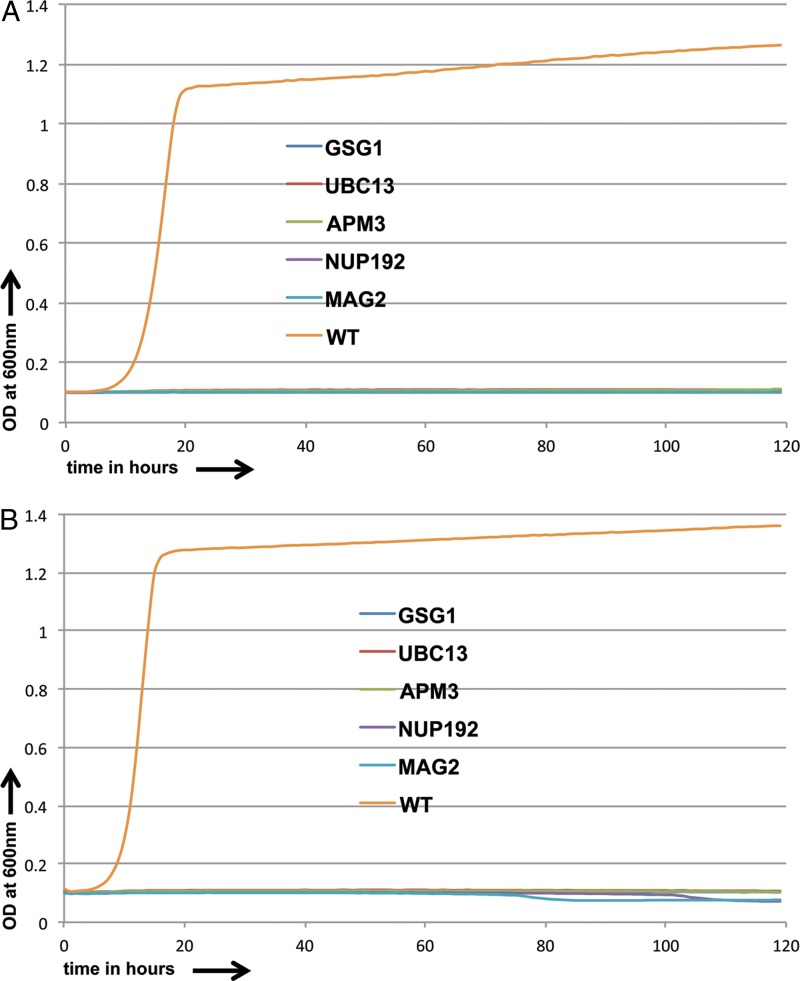
Growth curves for sensitive C. albicans strains and CaSS1 (WT) in the presence of caspofungin (0.2 μg/ml) (A) and anidulafungin (0.03 μg/ml) (B) over a 5-day period.

A set of 37 of the most sensitive of the 78 commonly sensitive strains were selected and tested at diminishing concentrations of both caspofungin (4 μg/ml, 2 μg/ml, and 1 μg/ml) and anidulafungin (0.1 μg/ml, 0.06 μg/ml, and 0.03 μg/ml). Overall, 19 strains were found to be sensitive to even the lowest drug concentration used for both echinocandins. Of these mutants, 14 showed sensitivity to one or more of the other drugs we tested. The remaining 5 mutated genes thus showed, among the drugs we tested, a specific sensitivity to the echinocandins. They included *GSG1*, encoding a putative subunit of the TRAPP complex involved in targeting of endoplasmic reticulum (ER)-to-Golgi transport vesicles (29); *UBC15*, encoding a putative E2 ubiquitin-conjugating enzyme (30); *APM3*, encoding a phosphorylated protein of unknown function (31) with upregulated transcripts in clinical isolates from HIV-positive patients with oral candidiasis (32); *NUP192*, which has orthologs encoding components representing a structural constituent of the nuclear pore (33), and *MAG2*, whose S. cerevisiae ortholog is implicated in DNA repair. All these inactivations caused clear sensitivity to the pair of echinocandins studied (Fig. 2A and B) but to none of the other drugs in our assays. Thus, both intracellular transport and DNA repair may be processes that can be modified to generate specific sensitivity to the tested echinocandins (reviewed in reference 34).

We determined the 50% inhibitory concentrations (IC_50_s) of caspofungin and anidulafungin for these mutant strains (Table 1). For both drugs, the sensitive strains showed enhanced IC_50_ values; the strains showed 40- to 50-fold enhanced sensitivity to caspofungin and 1.5- to 10-fold enhanced sensitivity to anidulafungin.

**TABLE 1:** IC_50_ values for all drugs for both sensitive and resistant strains

Drug and strain sensitivity	IC_50_ (μM) for indicated WT or mutant strain^*a*^					
Caspofungin						
Sensitive strains	WT	*GSG1*	*UBC13*	*APM3*	*MAG2*	*NUP19*
	0.072	0.0013	0.0015	0.0018	0.0015	0.0015
Resistant strains	WT	*DFG5*	*GPI12*	*MSU1*	*ITR1*	*ALO1*
	0.072	0.24	0.23	0.25	0.18	0.33
Anidulafungin						
Sensitive strains	WT	*GSG1*	*UBC13*	*APM3*	*MAG2*	*NUP192*
	0.013	0.0087	0.0077	0.0093	0.0013	0.0083
Resistant strains	WT	*DFG5*	*GPI12*	*MSU1*	*ITR1*	*ALO1*
	0.013	0.023	0.017	0.025	0.02	0.013
Fluconazole						
Sensitive strains	WT	*SEC65*	*NPY1*	*PAA11*	*SOG2*	*ERG251*
	23	12	7.2	5.4	3.7	2.2
Resistant strains	WT	*ERG3*	*HCS1*	*SLD1*	*RAP1*	*ADP1*
	23	34	30.03	32	54	37
Posaconazole						
Sensitive strains	WT	*SEC65*	*NPY1*	*PAA11*	*SOG2*	*ERG251*
	0.13	0.099	0.1	0.1	0.085	0.089
Resistant strains	WT	*ERG3*	*HCS1*	*SLD1*	*RAP1*	*ADP1*
	0.13	0.27	0.37	0.24	0.25	0.24
Amphotericin B						
Sensitive strains	WT	*PDS5*	*PCM1*	*RPO21*	*MNR2*	*YAK1*
	0.52	0.28	0.35	0.24	0.18	0.21
GPI-C107						
Sensitive strains	WT	*ERG6*	*NCP1*	*CHS7*	*PAT1*	*LEM3*
	1.9	0.32	0.058	0.16	0.056	0.11
Resistant strains	WT	*PEX14*	*CDC1*	*SNG1*	*ERP3*	*POR1*
	1.9	2.9	4.1	3.7	3.4	4.7
GPI-Ly7						
Sensitive strains	WT	*ERG6*	*NCP1*	*CHS7*	*PAT1*	*LEM3*
	13	2.7	2	0.93	1.7	1.2
Resistant strains	WT	*PEX14*	*CDC1*	*SNG1*	*ERP3*	*POR1*
	13	31	31	64	21	26

a The values typically fall within the same ranges as those for previously published values for the same drugs (82–86).

Using assays similar to ours, previous studies identified inactivations of genes that caused echinocandin sensitivity. Several of these genes (*SSU81*, *MSB2*, *VPS28*, and *CEK1*) were also identified in our screen as causing echinocandin sensitivity (but not hypersensitivity) when inactivated. Other genes identified in the literature, such as *MID1*, *MNN1*, and *RIM101*, affected other drug sensitivities as well as caspofungin sensitivity and thus were not uniquely influencing the echinocandin response. However, *CDC10*, whose deletion specifically caused increased sensitivity to caspofungin (35), was not picked up in our screen, although the mutant is in the GRACE 1.0 collection.

**FIG 3:**
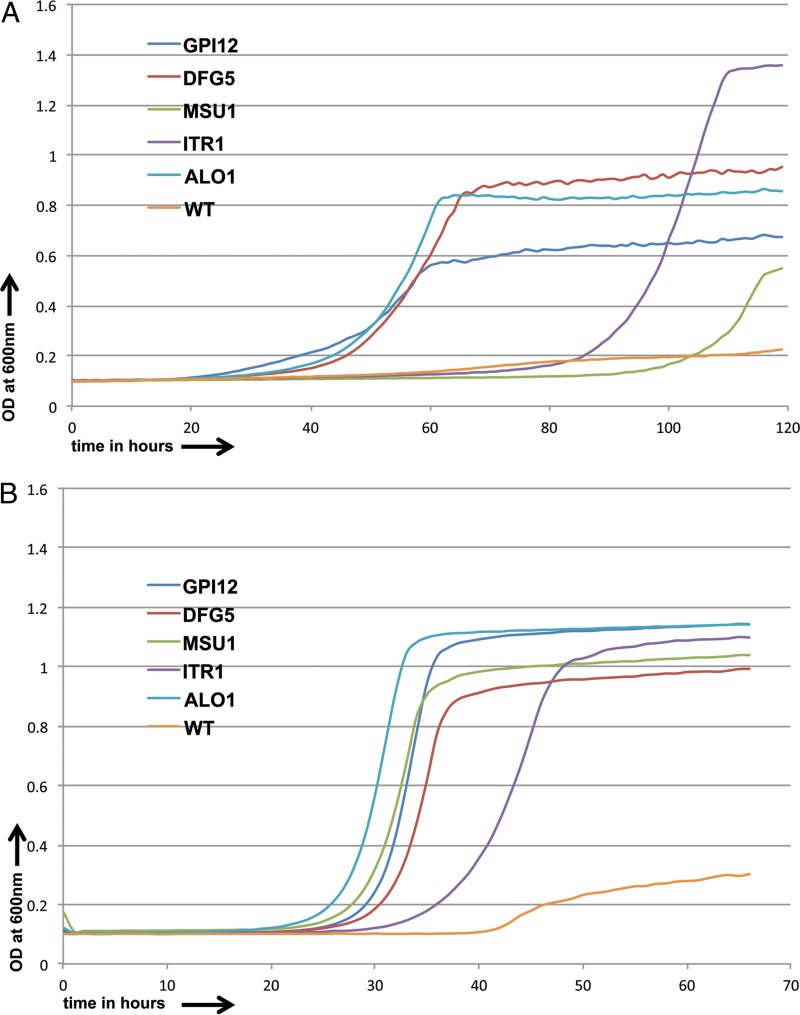
Growth curves for resistant C. albicans strains and CaSS1 (WT) in the presence of caspofungin (1.5 μg/ml) (A) and anidulafungin (0.1 μg/ml) (B) over 5- and 2.8-day periods, respectively.

We also tested the 37 most resistant strains among the 39 commonly resistant strains identified in our screen with incrementally increasing concentrations of caspofungin (4 μg/ml, 6 μg/ml, and 8 μg/ml) and anidulafungin (0.1 μg/ml, 0.15 μg/ml, and 0.2 μg/ml). We identified 12 strains that were resistant to even the highest drug concentrations used. Among them, the strains disrupted for *CRH11*, *ADP1*, and *PCL6* also showed resistance to other drugs, so there were nine strains that showed consistent resistance to both echinocandins but not to the other compounds investigated; five of the most resistant among them were investigated more thoroughly (Fig. 3A and B). These included strains with mutations of *DFG5*, which encodes an N-linked mannoprotein that is predicted to be a cell wall component; *GPI12*, whose S. cerevisiae ortholog encodes an ER membrane protein implicated in the second step of glycosylphosphatidylinositol (GPI) anchor assembly: *MSU1*, whose product is a mitochondrial exoribonuclease involved in mitochondrial rRNA quality control; *ITR1*, encoding a putative MFS glucose/myoinositol transporter; and *ALO1*, encoding a plasma membrane-localized d-arabinono-1,4-lactone oxidase.

We determined the IC_50_s of both drugs for the wild-type (WT) and mutant strains (Table 1) and found that for both drugs, the IC_50_ values for the majority of the resistant strains were 2- to 5-fold higher than those for the wild-type strain.

We compared the genes identified in our screen with genes identified in the literature as causing significant resistance to growth in the presence of echinocandins when disrupted. *DFG5* had previously been identified as conferring specific caspofungin resistance when inactivated (36). Intriguingly, although we did not identify *PGA4* as a gene conferring specific echinocandin resistance in our assay as reported previously (36), we did find the disruption to cause sensitivity to the pyridine amide C107. Since pyridine amides target GPI anchor protein production, this overlap is interesting.

### Azoles.

**FIG 4:**
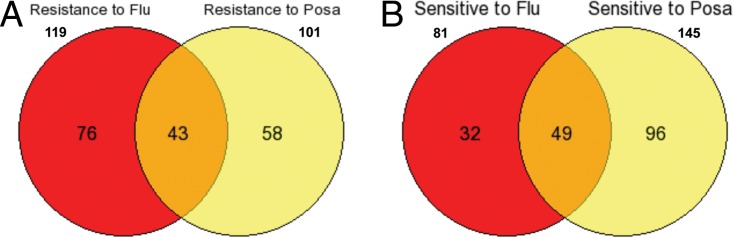
Comparison of genes showing sensitivity and resistance to fluconazole (Flu) and posaconazole (Posa). (A) Forty-three genes showed resistance to both Flu and Posa. (B) Forty-nine genes showed sensitivity to both Flu and Posa.

Azoles are another key class of antifungal compounds. In contrast to the echinocandins, which disrupt wall biosynthesis, azoles attack the cell membrane by disrupting the synthesis of ergosterol (37). We investigated the actions of two azoles: fluconazole (Flu), a broad-spectrum azole, and posaconazole (Posa), an expanded-spectrum azole (38). As was also seen with the echinocandins, the profiles of the two azoles showed considerable overlap for both sensitivity and resistance (Fig. 4A and B). We identified 119 strains that were resistant to fluconazole and 101 strains resistant to posaconazole, with an overlap of 43 strains, and we found 81 strains sensitive to fluconazole and 145 strains sensitive to posaconazole, with an overlap of 49 strains.

**FIG 5:**
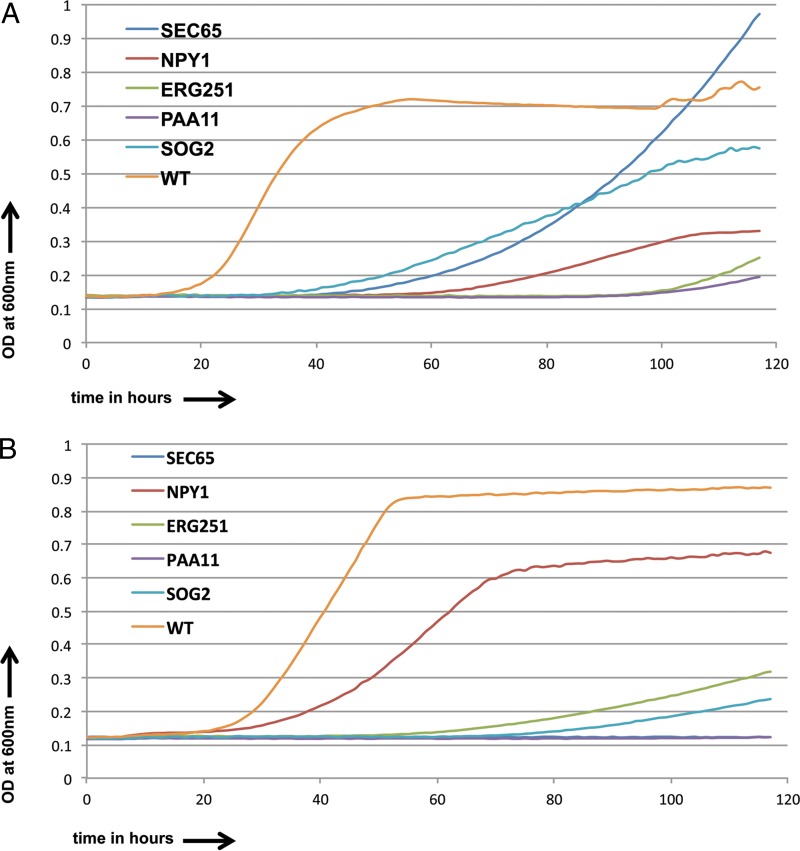
Growth curves for sensitive C. albicans strains and CaSS1 (WT) in the presence of fluconazole (3.0 μg/ml) (A) and posaconazole (0.07 μg/ml) (B) over a 5-day period.

A set of 37 of the most sensitive of the 49 commonly sensitive strains were selected and tested at diminishing concentrations of both fluconazole (10 μg/ml, 7 μg/ml, and 3 μg/ml) and posaconazole (0.1 μg/ml, 0.07 μg/ml, and 0.03 μg/ml). Overall, 5 strains were found to be sensitive to even the lowest drug concentration used for both azoles. These included strains with mutations of *SEC65*, encoding a component of the protein-targeting signal recognition particle (SRP); *NPY1*, encoding a putative NAD^+^ diphosphatase; *ERG251*, encoding a C-4 sterol methyl oxidase in the ergosterol pathway; *PAA11*, encoding a putative polyamine acetyltransferase; and *SOG2*, encoding a leucine-rich repeat domain-containing protein of the RAM cell wall integrity signaling network. The growth curves for these strains compared to those for the control in the presence of fluconazole and posaconazole are shown in Fig. 5A and B. We also calculated the IC_50_ values (Table 1) for these strains and found that compared to those for the wild type, the IC_50_ values were 2- to 3-fold enhanced for fluconazole and 1.2- to 1.5-fold enhanced for posaconazole.

Of the genes reported in the literature whose deletion causes sensitivity to azoles, several are found in the GRACE 1.0 collection. Six of these previously reported genes (*SOG2*, *CCH1*, *PTC2*, *BCR1*, *RPN4*, and *GZF3*) were picked up in our screen. *VPS28*, which was reported in the literature as causing sensitivity to caspofungin, amphotericin B, and fluconazole upon deletion (39), was observed in our screen to cause sensitivity to caspofungin, amphotericin B, and pyridine amides but not fluconazole. However, *SWI4*, whose deletion was reported in the literature as giving strong reproducible sensitivity to azoles, was not identified in our screen as conferring azole sensitivity but was seen (40) to cause echinocandin sensitivity.

**FIG 6:**
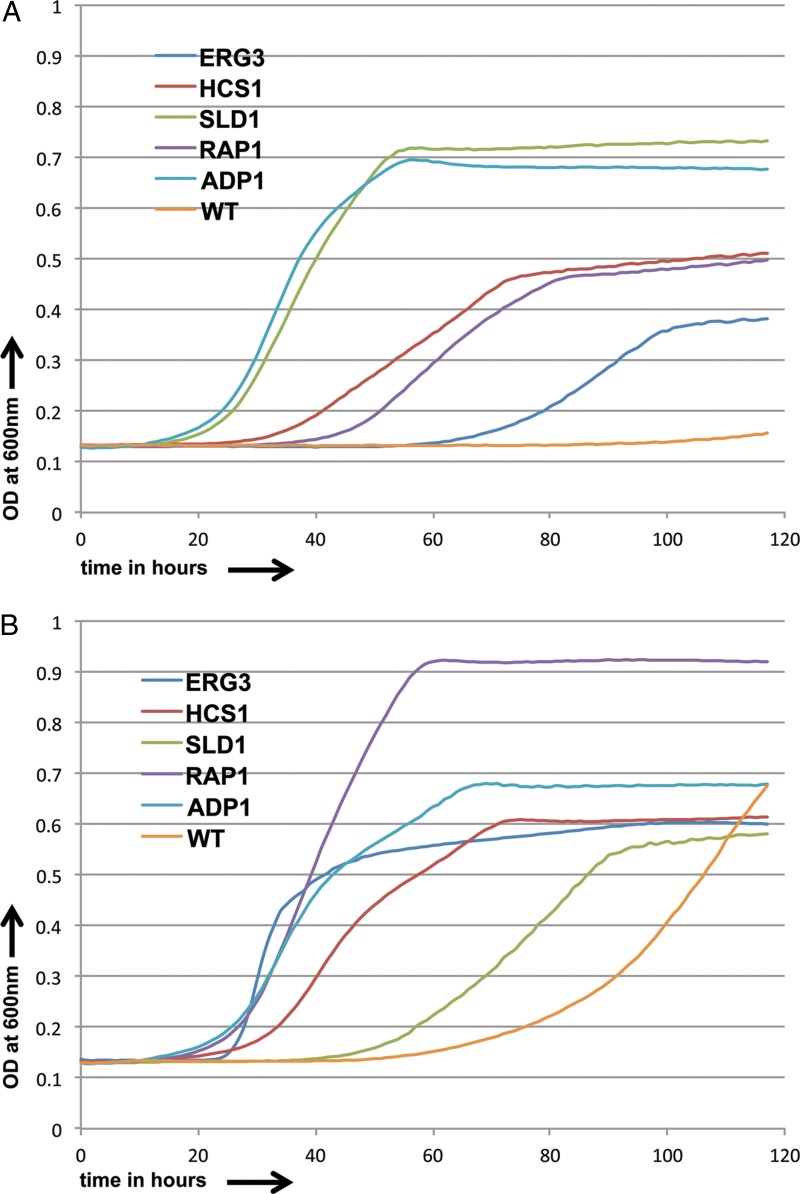
Growth curves for resistant C. albicans strains and CaSS1 (WT) in the presence of fluconazole (15.0 μg/ml) (A) and posaconazole (1.0 μg/ml) (B) over a period of 5 days.

The 37 most resistant strains among the 43 commonly resistant strains were also tested with incrementally increasing concentrations of fluconazole (10 μg/ml, 15 μg/ml, and 20 μg/ml) and posaconazole (0.1 μg/ml, 0.15 μg/ml, and 0.2 μg/ml). We identified 5 mutant strains that were resistant to even the highest drug concentrations used. Among them were strains with disruptions of *ERG3*, encoding a C-5 sterol desaturase involved in the ergosterol pathway; *HCS1*, encoding a putative ATP-dependent 5′-3′ DNA helicase; *SLD1*, encoding a sphingolipid delta-8 desaturase; *RAP1*, encoding a multifunctional transcription factor controlling telomeres and ribosomal proteins; and *ADP1*, the gene for a putative PDR subfamily ABC transporter. The growth curves are shown in Fig. 6A and B.

We next calculated the IC_50_ values (Table 1) for the genes whose deletion caused resistance and found that for fluconazole, the values changed 1.3- to 1.6-fold, while for posaconazole they changed 1.5- to 3-fold. There are relatively few genes reported in the literature whose deletion causes strong resistance to azoles; all candidates in the literature that are also found in the GRACE 1.0 collection (*SCH9*, *ERG3*, and *ERG6*) were observed in our screen.

### Amphotericin B.

**FIG 7:**
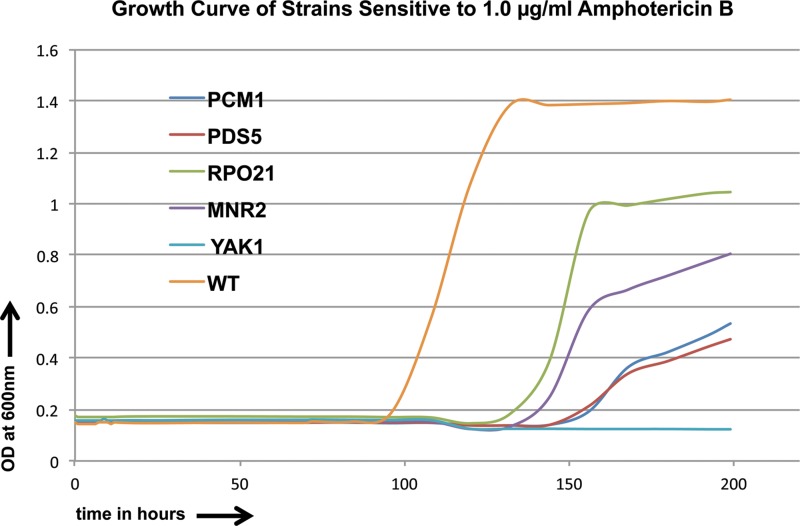
Growth curves for sensitive C. albicans strains and CaSS1 (WT) in the presence of amphotericin B (AmB) (1.0 μg/ml) over an 8-day period.

Amphotericin B is the most commonly used polyene antifungal drug. Its likely mode of action is to bind to ergosterol in the cell membrane, allowing leakage of cellular components and ultimately leading to the death of the cell. We profiled the spectrum of sensitivity and resistance of strains to this compound; no genes were found to confer convincing resistance to amphotericin B, while genes conferring sensitivity were common, with 268 disruption strains identified as being sensitive to the compound at 0.7 μg/ml and 1.0 μg/ml. We chose 5 strains that were highly sensitive to the drug at 0.7 μg/ml for further study. These strains were inactivated for *PCM1/AGM1*, encoding a putative phospho-acetylglucosamine mutase; *PDS5*, the gene for a protein with a predicted role in establishment and maintenance of sister chromatid condensation and cohesion; *RPO21*, encoding an RNA Pol II component; *MNR2*, encoding a putative ion transporter; and *YAK1*, encoding a predicted serine-threonine protein kinase. Growth curves for the supersensitive strains and the CaSS1 WT strain in the presence of AmB over 3.5 days are shown in Fig. 7. We further calculated the IC_50_ values (Table 1) for the strains with gene deletions that caused sensitivity and found that the IC_50_ values changed 1.5- to 3.0-fold for the strains tested.

Most of the genes reported in the literature as causing significant sensitivity to amphotericin B upon deletion and that are also in the GRACE 1.0 collection were detected in our screen, including *PDK2* (41), *BUB2* (41), *MET16* (41), *TPS1* (42), and *CYR1* (43). However, *FEN12* (44) was not seen as conferring AmB sensitivity in our assay, but it was found to cause resistance to azoles when disrupted.

### Pyridine amide derivatives.

**FIG 8:**
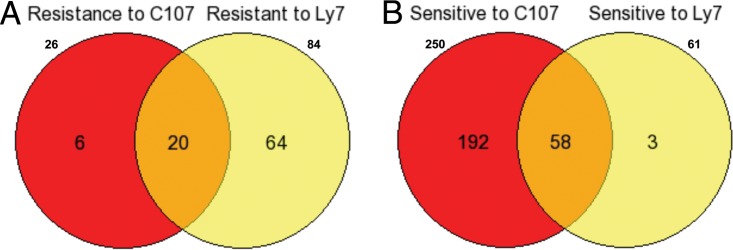
Comparisons of genes showing sensitivity and resistance to two E1210 derivatives (GPI-C107 and GPI-LY7). (A) Twenty strains showed a common resistance to both GPI-C107 and GPI-LY7. (B) Fifty-eight strains showed a common sensitivity to both GPI-C107 and GPI-LY7.

New antifungal drugs are interesting for both scientific and potential commercial purposes. The Nakamoto group reported that the pyridine amide 10b (45) and the 2-aminopyridine E1210 (46) inhibited the function of the Gwt1 protein in the GPI biosynthetic pathway and exhibited good bioactivity against Candida albicans (47) and Aspergillus fumigatus. A series of analogs of compounds 10b and E1210 have been synthesized and were found to display broad-spectrum antifungal activity and even to inhibit fluconazole-resistant Candida albicans (48). Two compounds (from Yan Li and Dazhi Zhang) of this family (GPI-C107 and GPI-LY7) were tested with the GRACE 1.0 collection to identify possible sensitive and resistant strains; both compounds had very similar profiles but were clearly distinct from the azoles, the echinocandins, and amphotericin B. As shown in Fig. 8B, the sensitivity profiles of these two pyridine amide compounds had clear overlap. Two hundred fifty strains showed sensitivity to GPI-C107, 61 strains showed sensitivity to GPI-LY7, and there were 58 strains in common (Fig. 8B). Similarly, 84 strains were found to show resistance to GPI-LY7, 26 strains showed resistance to GPI-C107, and 20 strains showed a common resistance to both compounds (Fig. 8A). These results suggest that the C107 compound has greater bioactivity than that of LY7, but the two compounds have otherwise essentially identical functions.

**FIG 9:**
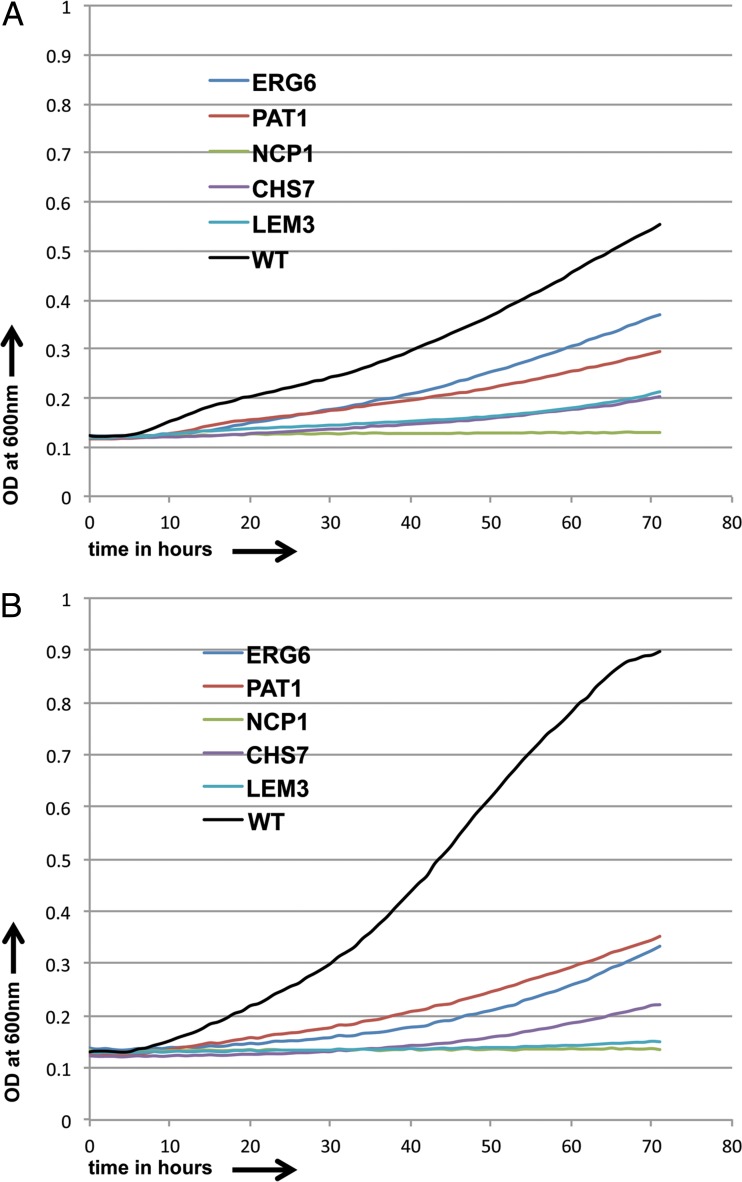
Growth curves for sensitive C. albicans strains and CaSS1 (WT) in the presence of GPI-C107 (0.25 μg/ml) (A) and GPI-LY7 (1.5 μg/ml) (B) over a period of 3 days.

The set of 58 strains sensitive to both pyridine amide derivatives was selected and tested at diminishing concentrations of both GPI-C107 (0.5 μg/ml and 0.25 μg/ml) and GPI-LY7 (2 μg/ml and 1.5 μg/ml). Five strains were found to be sensitive to even the lowest drug concentration used for both pyridine amide derivatives (Fig. 9A and B), and those strains were deleted for *ERG6*, *NCP1*, *CHS7*, *PAT1*, and *LEM3* (49). *ERG6* encodes a delta-(24)-sterol C-methyltransferase involved in the ergosterol biosynthesis pathway. *NCP1* encodes an NADPH-cytochrome P450 reductase that acts with Erg11p in sterol 14-alpha-demethylation in ergosterol biosynthesis. *CHS7* encodes a key regulator of wild-type chitin synthase III activity (*CHS3*). *PAT1* has orthologs that encode chromatin and mRNA binding activity, and *LEM3* encodes a putative cell surface receptor protein. Thus, the genes whose deletion caused high sensitivity seem to come from either the ergosterol synthesis pathway or be involved in the synthesis of the cell wall and chitin.

Furthermore, we calculated IC_50_ values (Table 1) for the two drugs for the genes whose deletion caused sensitivity and found that for GPI-C107, the IC_50_ value changed 6- to 34-fold for the strains, while for GPI-LY7, the value changed 5- to 15-fold.

**FIG 10:**
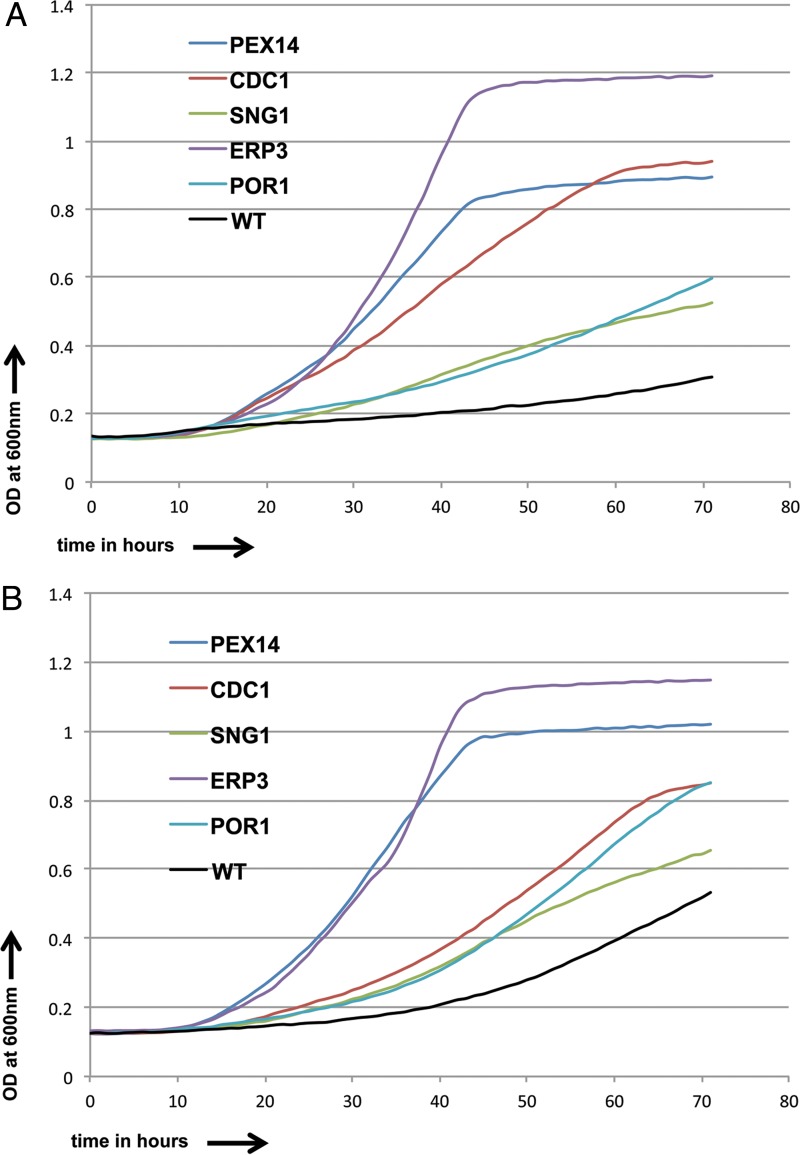
Growth curves for resistant C. albicans strains and CaSS1 (WT) in the presence of GPI-C107 (2.0 μg/ml) (A) and GPI-LY7 (8.0 μg/ml) (B) over a period of 3 days.

The 20 strains commonly resistant to both pyridine amide derivatives were tested with incrementally increasing concentrations of GPI-C107 (0.5 μg/ml, 1 μg/ml, and 2 μg/ml) and GPI-LY7 (2 μg/ml, 4 μg/ml, and 8 μg/ml). Five strains were found to be resistant to even the highest drug concentration for both pyridine amide derivatives and were defective in *PEX14*, *CDC1*, *SNG1*, *ERP3*, and *POR1* (Fig. 10A and B) (49). *PEX14* is an open reading frame (ORF) with orthologs whose products bind to peroxisome matrix targeting signals 1 and 2. *CDC1* encodes a putative protein involved in GPI anchor remodeling prior to the attachment of cell wall proteins to beta-1,3-glucan, removing ethanolamine phosphate from the first mannose of the GPI anchor, along with being a lipid phosphatase of the endoplasmic reticulum with roles in DNA repair, actin cytoskeleton organization, and cellular manganese ion homeostasis. *SNG1* is an ORF with orthologs encoding plasma membrane-localized products with roles in nucleobase-containing compound transport. *ERP3* is an uncharacterized ORF with orthologs encoding Golgi apparatus and endoplasmic reticulum localization functions. *POR1* encodes a mitochondrial outer membrane porin present in the detergent-resistant membrane fraction and is a possible lipid raft component. Thus, genes whose deletion causes pyridine amide resistance have wide-ranging functions. When the IC_50_ values were calculated for the drugs in the mutant strains, the observed values were seen to vary 1.5- to 2.5-fold for both drugs.

### Relationships among general resistance genes and general sensitivity genes.

All 4 classes of compounds (azoles, echinocandins, polyenes, and pyridine amides) have different molecular targets, so it was of interest to see if there were classes of genes that were found to be involved in sensitivity or resistance to multiple classes of compounds.

**FIG 11:**
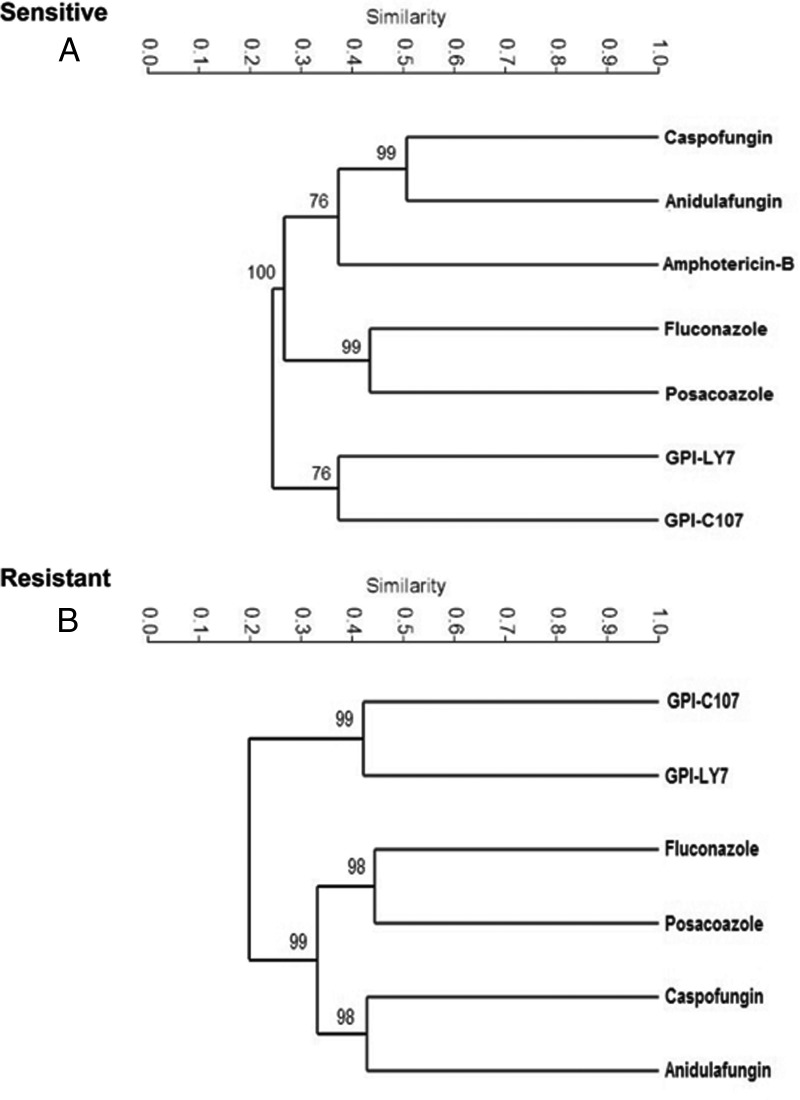
Dendrograms showing the results of a hierarchical cluster analysis based on sensitivity (A) and resistance (B) among C. albicans strains. The dendrograms illustrate the similarity between the classes of antifungal drugs tested. Each of the functionally related compounds mapped closely in the clustering diagram.

We initially used hierarchical clustering to compare the data sets for all the compounds tested. As shown in Fig. 11, each of the functionally related compounds mapped closely together in the dendrogram. The pyridine amide compounds were clustered distinctly from the other compounds when assessed for both resistance and sensitivity. Interestingly, amphotericin B clustered somewhat with the echinocandins in the sensitivity assay.

We next compared the genes implicated in general resistance to azoles, echinocandins, and the pyridine amide derivatives. No mutations in the set of genes we examined conferred resistance to all classes of drugs, and in fact only two genes, *YHB5* (encoding a flavo-hemoglobin-related protein) and *PCL7* (encoding a putative cyclin), showed up for more than two classes; mutations in these genes caused resistance to azoles, echinocandins, and the pyridine amide derivatives. There was a small set of genes whose inactivation led to resistance for both echinocandins and azoles; in addition to *YHB5* and *PCL7*, we identified the following six other genes: *TLG2*, encoding a t-snare protein; *CHR11*, encoding a GPI-anchored transglycosylase; *ORF19.3329*, involved in calcium-mediated signaling; *ADP1*, encoding a putative transporter; *ORF19.6185*, encoding a candidate pseudouridine glycosidase; and *ORF19.6199*, encoding a potential DNA helicase. Therefore, overall, the gene sets implicated in drug resistance among the compounds were essentially distinct, consistent with their different cellular targets, and among the genes that were implicated in resistance to more than one drug class, there was no evidence of a specific functional class.

In the case of genes whose inactivation caused drug sensitivity, no genes were identified that conferred a general sensitivity to all the drugs, but several genes were found to cause sensitivity to three classes at the same time. The genes whose inactivation led to sensitivities to the pyridine amide derivatives as well as the echinocandins and amphotericin B included putative cytoskeletal element genes, such as *SAC7*, *MEA1*, and *MYO1*, as well as genes encoding candidate components of intracellular trafficking, such as *ORF19.3458*, *VPS24*, and *VPS28*. Several genes were also implicated in conferring sensitivity to echinocandins and azoles as well as amphotericin B; these genes had very diverse functions and included *POR1*, encoding a mitochondrial porin; *GIG1*, encoding a glucosamine-induced product; *CCH1*, encoding a calcium channel; *HRT2*, encoding transposition of ty3; *BUB2*, encoding a checkpoint GAP; *ORF19.6152*, encoding a mitochondrial protein; and *GNP3*, encoding a glutamine permease.

### Confirmations.

**FIG 12:**
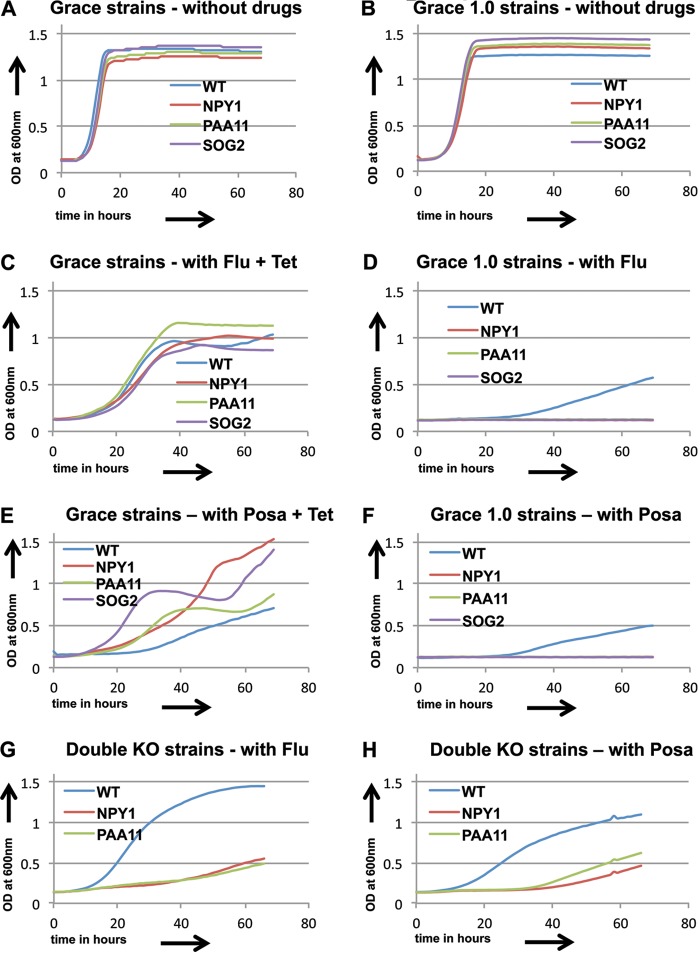
(A to F) Growth curves for three sensitive strains (*NPY1*, *PAA11*, and *SOG2*) compared to CaSS1 (WT) for the GRACE and GRACE 1.0 library strains show different results for growth in the presence of fluconazole or posaconazole. (G and H) Growth curves for *NPY1* and *PAA11* double knockout strains (double KO) and the wild-type strains (WT) in the presence of fluconazole (10 μg/ml) and posaconazole (0.1 μg/ml). The WT growth curve shows the average growth for the SN76 and SN148 wild-type strains.

Because of the appearance of unrelated loss-of-heterozygosity (LOH) events in a subset of the GRACE 1.0 library, we tested examples of the sensitive and resistant classes for each drug with the original GRACE strains under tetracycline inactivation conditions. These assays confirmed the sensitivity and resistance profiles for the genes conferring sensitivity or resistance to the echinocandins, pyridine amides, and amphotericin B. However, the combination of tetracycline and azoles was found to give anomalous results—several of the GRACE strains inactivated by tetracycline gave results that were inconsistent with the transactivator-deleted GRACE 1.0 strains (Fig. 12). In these cases, we investigated null mutants of the genes (*SOG2*, *NPY1*, and *PAA11*) to test the behavior of the inactivating mutations. Previous work by others, using C. albicans strain knockout experiments, demonstrated a sensitivity profile for *SOG2* mutants in the presence of azoles similar to that observed with the GRACE 1.0 library (50). A double knockout (KO) of the *NPY1* gene of strain SN76 was generated using Arg4 and His1 selection by use of pFA-Arg4 and pFA-His1 (51). The disruption strain generated was tested along with strain SN76 by use of 10 μg/ml fluconazole (Flu) (Fig. 12G) and 0.1 μg/ml posaconazole (Posa) (Fig. 12H) over a 3-day period, and it showed sensitivity to both azoles compared to that of SN76 (WT). A *PAA11* double KO mutant in the SN148 background strain was generated using clustered regularly interspaced short palindromic repeat (CRISPR) technology (52) and, together with the WT SN148 strain, was tested in the presence of 10 μg/ml Flu (Fig. 12G) and 0.1 μg/ml Posa (Fig. 12H) over a 3-day period. In the figure, the WT curve represents the average growth of the WT SN76 and WT SN148 strains. In all three cases, the null phenotype agreed with that of the GRACE 1.0 strain, suggesting that the presence of tetracycline in the GRACE strain assay was generating inconsistent results.

### Comparisons with other data sets.

**FIG 13:**
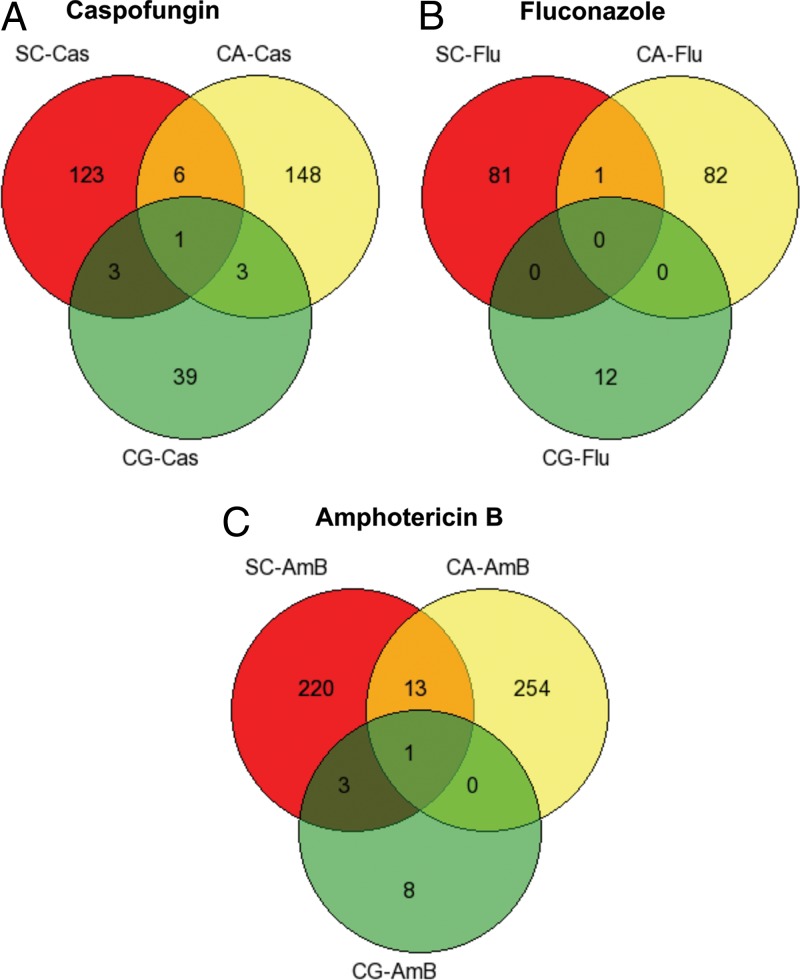
Overlap in genes causing sensitivity in three species, i.e., S. cerevisiae (SC), C. albicans (CA), and C. glabrata (CG), to the presence of caspofungin (A), fluconazole (B), or amphotericin B (C).

Similar library screens for sensitivity to antifungal drugs have been performed with other fungi. In particular, the comprehensive disruption collection available for the budding yeast S. cerevisiae (14, 53) has been exploited to investigate the sensitivity profile for caspofungin and fluconazole, and recently, an extensive collection of disruption strains of the fungal pathogen Candida glabrata was examined to identify the roles of specific genes in causing sensitivity to azoles and echinocandins (18). A comparison of the collection of genes found in the GRACE 1.0 collection with the S. cerevisiae genome identified that 763 of the 887 disruptions had a clear ortholog in the yeast genome. Similarly, comparison of the 887 C. albicans disruption strains identified 464 orthologs in the collection of 557 disruptions characterized for C. glabrata. We compared the list of genes whose inactivation caused sensitivity to caspofungin, fluconazole, and amphotericin B in S. cerevisiae and C. glabrata with the ones found in C. albicans and show the data as a set of Venn diagrams in Fig. 13. As can be seen, the amount of overlap is very limited.

## DISCUSSION

The relationship between genes and drugs is important for both academic and practical reasons. In the case of antifungal drugs targeting C. albicans, it is of interest to identify both the genetic strategies the organism can exploit to escape current therapeutic approaches and potential genetic synergies with currently used drugs. The dose sensitivity curves for all the drugs are shown in Fig. S2 in the supplemental material.

In the present study, we investigated the interactions of a set of approximately 900 null mutations of nonessential genes of the fungal pathogen C. albicans with a set of antifungal drugs. This work identified three broad patterns in the relationships among the null mutants and the drugs. The first is that drugs in the same antifungal drug class generate similar patterns of resistance and sensitivity; the second is that drugs of different classes and with different intracellular targets identify different sets of genes conferring sensitivity or resistance. Neither of these observations is unexpected but simply serves to underline the point that there is a genetic basis for the physiological response to antifungal compounds. The third broad pattern is, however, more surprising: there is very little correspondence in the genes conferring sensitivity to each drug class among related fungi. Even though, for example, the ergosterol pathway is the target of azoles in the related ascomycetes C. albicans, C. glabrata, and S. cerevisiae, among these three species the null mutations that confer heightened sensitivity to these compounds are very different.

There are different possible explanations for this observation. One is that the level of functional redundancy varies significantly among the species. C. albicans is a pre-whole-genome-duplication yeast, whereas S. cerevisiae and C. glabrata have undergone the duplication (54, 55). Essential genes differ considerably between C. albicans and S. cerevisiae, even though the metabolic circuitries are quite similar between the two organisms (56). This suggests differences in functional redundancy for essential genes as well. These potential differences in redundancy do not appear to be driven simply by the pre- and post-whole-genome-duplication patterns, because the differences between C. albicans and C. glabrata are not dramatically greater than those between S. cerevisiae and C. glabrata.

A second mechanism that may lead to different spectra of genes implicated in drug sensitivity or resistance involves the differences in the overall wiring of the response circuits. Large-scale rewiring of transcriptional circuits has occurred between these species (57, 58), and the rewiring of posttranslational modifications can generate major differences in metabolic responses (57, 59, 60). These observations argue that the genetic impact of loss of particular genes may depend in part on their relative importance in the specific circuits that have evolved in each separate fungus. Further work, including the construction of double mutant combinations among the genes conferring drug sensitivity or resistance, will be necessary to map out whether there are related genetic circuits whose critical elements differ between species or whether the entire circuitry is different among the different species.

While we currently have a number of therapeutic interventions for fungal diseases, there is a continuing interest in identifying and characterizing new antifungal drugs. We therefore looked at the patterns of sensitivity and resistance generated by compounds of the pyridine amide class of molecules, which have been proposed to target the biosynthesis of GPI anchors through inhibition of Gwt1 (61), a target distinct from those of the clinically available echinocandins, azoles, and amphotericin B. Pyridine amides have been seen to have good anti-Candida and anti-Aspergillus profiles *in vitro* (45). Recently, more members of this class of compounds were synthesized and shown to have bioactivity against a variety of fungi, including C. albicans, C. parapsilosis, C. glabrata, and Cryptococcus neoformans. Selected members of this group used against C. albicans caused destruction of the mannoprotein coat, consistent with inhibition of the biosynthesis of GPI anchor proteins (48). The two pyridine amide compounds tested here were found to generate very similar profiles, strongly supporting the hypothesis that the two molecules target the same cellular process. The overall spectrum of resistance- and sensitivity-generating mutations was also very different from that shown for the currently used drugs, supporting the idea that the pyridine amides hit a target distinct from that of the azoles, echinocandins, or polyamines. Intriguingly, however, of the five genes whose deletion causes hypersensitivity, two are related to ergosterol biosynthesis and two are related to the cell wall, which represent the respective targets of the azoles and the echinocandins. A further interesting observation is that one deletion that causes resistance is in a gene encoding a protein involved in GPI anchor remodeling; loss of this function potentially compensates for the inactivation of Gwt1 by the drug. Initial studies in our lab identified possible complications in the investigation of azole responses by use of the GRACE conditional mutant collection and led in part to the interest in creating the nonconditional derivative library. These complications may come about in part because of the ability of tetracycline or doxycycline to chelate iron (24) or because of a loss of mitochondrial function due to the presence of tetracycline (62). This issue seemed to be of concern primarily for the azole component of our study—3 of 5 genes identified as conferring azole resistance when inactivated in the derivative library were not confirmed by tetracycline-based inactivation of the target gene in the GRACE collection strain. All three discrepant genes were tested by use of null mutants, either created by us (*NPY1* and *PAA11*) or identified in the literature (*SOG2*) (50), and in each case the null mutant confirmed the phenotype of the derivative library mutant, suggesting that the GRACE strain shutoff phenotype did not represent the response of a null mutant to azole treatment. None of the other drug classes showed a discrepancy between the tetracycline-repressed GRACE library phenotype and the GRACE 1.0 derivative collection phenotype.

Somewhat surprisingly, analysis of the genes influencing azole sensitivity and resistance identified members of the ergosterol biosynthetic pathway that had opposing effects on the azole response. Deletion of *ERG3* led to azole resistance, and deletion of *ERG251/ERG25* caused sensitivity. *ERG3* encodes the Δ5,6-desaturase acting late in the ergosterol biosynthesis pathway (63). Erg3p is believed to be responsible for converting tolerated 14-methyl intermediates, which accumulate because of azole inhibition of 14C-lanosterol demethylase, into the toxic sterol 14-methylergosta-8,24(28)-dien-3,6-diol(4,5). Therefore, *ERG3* inactivation confers azole resistance because the toxic sterol is not formed in its absence, even though the biosynthetic pathway is inhibited by the presence of the azole.

The explanation for sensitivity generated by deletion of *ERG251/ERG25* is less obvious, and in fact, *ERG25* deletion in S. cerevisiae confers resistance to cells in the presence of azoles, along with reduced levels of heme (64). It is not clear if these *ERG25*-deleted cells are viable in the presence of azoles without any other mutations. *ERG25* shows only modest overexpression in the presence of ketoconazole (65). Intriguingly, in C. albicans, *ERG25* expression is highly upregulated (>10-fold) by the presence of fluconazole both in yeast cultures and in biofilms (65, 66). This implies that in the presence of azoles, C. albicans cells need *ERG25* to survive. Further, the function of Erg25 is to produce zymosterol from 4,4-dimethylzymosterol (67). It has been observed that 4,4-dimethylzymosterol is not a tolerated sterol in cells and that its accumulation causes cells to die (67). Thus, in a C. albicans *ERG25*-deleted background with no other mutations, it is highly probable that the strain would be sensitive to azoles.

The finding of no AmB-resistant strains in our study is supported by previous studies, which have also failed to show good candidates for gene inactivations causing AmB resistance (68, 69). The mechanism of action of this macrolide drug has been thought to be binding to ergosterol, the major sterol of the cell membrane, leading to formation of small pores and thus increasing permeability for protons and monovalent cations, such as Na, and depolarization of the membrane (70). However, there is also recent evidence that cell death may not be linked to the formation of these pores and that the primary mechanism of amphotericin B killing is through binding with ergosterol (71–73). There is also evidence of mechanisms involving auto-oxidation and generation of reactive oxygen species (ROS) and free radicals in the cell (74, 75), and it has been speculated that AmB-induced accumulation of ROS can possibly explain the low rate of resistance to AmB. Certainly, if there are many independent pathways for AmB function in fungal killing, it is not surprising that a single gene inactivation is unable to produce a clear resistance phenotype.

An interest in improving antifungal therapies requires that we understand the mechanisms by which current therapies function and how the target can block or bypass these therapies. In this study, we used chemogenomics to investigate the genetic networks in C. albicans that underlie sensitivity or resistance to a number of current or potential antifungal drugs. Genes whose deletion enhances resistance to drugs may provide escape routes to cells challenged with the drug, and genes whose deletion enhances sensitivity to a drug can provide insight into potential synergizing strategies. In the future, double mutant combinations of genes separately capable of leading to drug sensitivity or resistance will allow us to establish the epistatic relationships among genes and to map out gene networks implicated in drug sensitivity or resistance. While we found that related drugs had similar gene profiles leading to resistance or sensitivity for a given fungal species, we also found that these profiles were surprisingly different among the different ascomycete fungi that have been examined systematically for gene-drug interactions. This points out that even when the actual drug target is the same among different fungi, the links between the drug target and the metabolic circuitry of each fungus can be different and complex.

## MATERIALS AND METHODS

### Media.

Strains were routinely grown in liquid YPD plus uridine (1% yeast extract, 2% peptone, 2% glucose, and 50 mg/liter uridine) at 30°C; screening was done in 200 μl YPD with specific concentrations of each compound. Fluconazole (Sigma) was added to 10 μg/ml from a 5-mg/ml stock in dimethyl sulfoxide (DMSO). Posaconazole (Sigma) was added to 0.1 μg/ml from a 2-mg/ml stock in methanol. Amphotericin B (MP) was added to 1 μg/ml from a 20-mg/ml stock in DMSO. Caspofungin (a gift from Merck) was added to 4 μg/ml from a 10-mg/ml stock in DMSO. Anidulafungin (a gift from T. Dahms) was added to 0.1 μg/ml from a 10-mg/ml stock in DMSO. GPI-C107 was added to 0.5 μg/ml from a 6.4-mg/ml stock in DMSO. GPI-LY7 was added to 2 μg/ml from a 6.4-mg/ml stock in DMSO. Control growth was done in liquid YPD with the corresponding solvent for each compound but lacking the compound. Liquid transfers to 96-well plates involved 200-μl samples transferred using a Corning multichannel pipette.

### Strains and plasmids.

The GRACE library of 2,425 strains and the WT strain for the collection, CaSS1 (20), were obtained from Merck. SN76 (76) was used for the construction of knockout strains, with *HIS1* and *ARG4* cassettes used for the *NPY1* double knockout (KO). Briefly, primers NPY1-S1 and NPY1-S2 (see Table S1 in the supplemental material) were used to PCR amplify the disruption cassettes from plasmids pFA-His1 and pFA-ARG4 (51), respectively, and the PCR products derived from pFA-His1 and pFA-Arg4 (77) were used to transform C. albicans strain SN76 to obtain the SN76 *NPY1* double knockout strain; plasmid pDH8 was used as the positive control for cassette amplification. Q5 high-fidelity polymerase (NEB) was used for PCR amplification. All oligonucleotides for construction and confirmation are listed in Table S1 in the supplemental material.

The SN148 *PAA11* (potential polyamine *N*-acetyltransferase) double KO strain was constructed using CRISPR, as previously documented (52). Briefly, phosphorylated and annealed *PAA11* guide RNA primers (LR182F and LR182R) (Table S1) were ligated to a ciprofloxacin (CIP)-treated, BsmBI-digested pV1093 vector. A mutagenic double-stranded oligonucleotide (LR183F and 183R) was used as a repair DNA. This oligonucleotide is complementary to *PAA11* and contains a mutation of the PAM sequence, two premature stop codons (UAA and UAG), and a HindIII restriction site. Standard lithium acetate transformation (78) was done on the SN148 strain (76). Transformants obtained on YPD nourseothricin plates were screened by PCR using primers LR184F and LR184R, followed by HindIII digestion. Correct clones were verified by sequencing. (The genotypes of all C. albicans strains used are given in Table S5.)

### Derivative library construction.

The GRACE 1.0 library for phenotype screening, mating ability identification, and drug target discovery was derived from the initial GRACE library (20) by selection against the *URA3* marker that was used to select for integration of the tetracycline transactivator cassette. The GRACE collection strains were inoculated in liquid YPD plus uridine and cultured at 30°C for 2 days. Each culture was then diluted 10^−1^ in sterile water, and 5-μl aliquots of each original culture and the 10^−1^ dilution were spotted on SD agar medium with 5-fluoroorotic acid (SD–5-FOA^+^) separately and cultured at 30°C for 6 days. For each strain, the dilution generating a few single colonies on the plate was identified, and a single colony was recultured in liquid YPD plus uridine for 3 days. The new YPD-plus-uridine culture was then diluted, sequential dilutions (10^−1^, 10^−2^, and 10^−3^) were spotted on YPD-plus-uridine agar and cultured at 30°C for 2 days, and then single colonies were chosen for each strain and tested for the ura^−^ phenotype. Finally, strains that failed to grow on SD−ura medium were collected from the corresponding YPD-plus-uridine agar plates and transferred to liquid YPD plus uridine to prepare the library stock. The original library stock was incubated at 30°C for 2 days and then replicated by robotic plating. After 2 days of incubation at 30°C, the new library stocks were mixed with 80% glycerol to final 20% glycerol-supplemented YPD cultures and were stored at −80°C in 96-well microtiter plates before use. All liquid media used for library construction were placed in 96-well microtiter plates, and all agar media used as described above were placed in rectangular petri dishes.

### Library validation.

Candidate strains in 96-well plates were plated on fresh YPD-uridine agar and incubated at 30°C for 2 days. Using a sterile 200-μl pipette tip, a single patch of each strain was transferred to a new well of a 96-well PCR plate, and the cells were dispersed in 25 μl of lysis buffer (12.5 μl of 10× PCR buffer [500 mM KCl, 100 mM Tris-HCl, pH 9.0, 15 mM MgCl_2_, 1% Triton X-100], 0.5 μl 50× lysis enzyme mix [20 U/μl; 50 mM Tris-HCl, pH 8.4, 100 mM KCl, 50% {vol/vol} glycerol, lyticase {260 U/mg; Sigma}], and 12 μl of MilliQ water) for extraction of DNA. PCR plates were incubated for 1 h at room temperature, and then 100 μl of MilliQ sterile water was added to each well. Plates were placed in a thermal cycler and heated at 95°C for 5 min in order to lyse cells and denature proteins. The PCR plates were then centrifuged at 3,000 rpm for 5 min (1,900 × *g*) in a Beckman Allegra X-12R centrifuge to pellet cellular debris. To test for the loss of the transactivator module by PCR, we used primers LR135F and LR135R, which amplify a 1.2-kb fragment of the Act1 promoter and the TetRGal4 activator. Ten microliters of DNA extracted as described above and rTaq polymerase were used in 50-μl PCR mixtures (a 10-nmol/ml concentration of each deoxynucleoside triphosphate [dNTP], a 10-nmol/ml concentration of each primer, 5 U/ml rTaq). PCR products were verified using 1% agarose gels.

In the end, we obtained 887 *ura3*^−^ 5-FOA-resistant strains that had lost the tetracycline transactivator and showed reasonable growth profiles. A list of the strains making up the GRACE 1.0 collection is provided in Table S2 in the supplemental material. We did LOH assays by testing the derivative strains for growth on −His plates and nourseothricin (clonNAT) plates because the 5-FOA selection process can result in a simultaneous loss of the *URA3* marker and loss of heterozygosity elsewhere in the genome (79). The two heterozygous markers, *HIS1* and the nourseothricin resistance marker, representing the two alleles of each random insertion generating the regulated gene disruption, were tested using SD−His and SD-nourseothricin^+^ media. Among all the mutants, 18.7% lost the *HIS* marker, 5.1% became nourseothricin sensitive, and no strains simultaneously lost both the *HIS1* and nourseothricin resistance markers. When these events were distributed over the C. albicans chromosomes, we observed that the distribution of markers was somewhat correlated with chromosome size; in general, the larger chromosomes (R, 1, and 2) showed higher frequencies of LOH than those of the smaller chromosomes (5 and 6) (Table S3).

### Drug screens and assays for IC_50_ determination.

The 887 strains of the GRACE 1.0 collection were arrayed in 17 96-well microtiter plates. The arrangement of the strains was designed to minimize artifacts due to effects of growth on the edges of plates and to provide an independent bar-coded confirmation for each plate. All the edges are occupied by the wild-type parent strain CaSS1, and each plate has a unique bar code for the last 6 inner wells (G6 to G11), consisting of various patterns of CaSS1 and the morphologically distinct mutant *dig1/dig1* strain (Fig. S1).

The GRACE 1.0 collection of strains was transferred to 96-well plates for analyses. Cells were inoculated from starting saturated culture plates into 200 μl of YPD by use of a replica pinning tool that transfers 2-μl samples and then by a further transfer from this intermediate dilution to wells containing 200 μl drug to generate a starting density that was an approximately 10^−4^ dilution of the saturated culture, and the cultures were grown at 30°C in YPD medium without shaking in the presence of the drug of interest for 120 h in initial screens and for 48 to 199 h in retests. The initial drug concentration for each compound was selected as that allowing saturated growth of the control strains after 3 days, while drug-free controls showed saturated growth in less than 2 days. Disruption strains that showed saturated growth before 3 days were designated resistant; strains that failed to grow or showed saturated growth after 4 days were designated sensitive. A semiquantitative scoring scale represented the day that saturated growth was observed, so scores of 1 and 2 represented resistance and those of 5 and 6 represented sensitivity. Each drug-library combination was repeated 3 times, and only strains that showed consistent sensitivity or resistance in all 3 replicates were designated such in the final data sets.

To calculate the IC_50_s of the various compounds, we used a modified version of the NCCLS M27 broth dilution method (80). Briefly, the sensitive and resistant C. albicans strains along with the wild-type control were grown overnight and diluted 1:100 in YPD, grown in 96-well microliter plates in YPD with eight different concentrations of the drugs, and tested in Tecan Sunrise machines with periodic shaking for 48 h at 30°C, with the optical density at 600 nm (OD_600_) measured every 15 min. Antifungal stock solutions were prepared in DMSO or methanol. Fourfold serial dilutions of the drugs were then prepared in DMSO or methanol in microcentrifuge tubes and stored at −20°C until use. For linear regression and computation of IC_50_ values, yeast growth from 36 to 40 h was used, and all tests were performed on three biological replicates. Endpoint readings were set as the antifungal concentrations causing at least 90% growth inhibition after 36 h of growth compared to the growth of the control. The IC_50_ was determined by linear regression analysis using Graph Pad Prism software (GraphPad Software Inc., San Diego, CA).

### Comparisons.

To assess the relationships among different antifungal drugs, a hierarchical cluster analysis based on resistance and sensitivity among C. albicans strains was performed using Bray-Curtis similarity with the paired-group algorithm as implemented in the software program PAST (81). The robustness of the clustering was determined through bootstrapping with 10,000 resamplings. The analysis was based on a binary matrix with gene names in columns and antifungal drugs in rows.

Recent publications (14, 18, 53) have shown the drug susceptibilities of members of gene deletion collections of S. cerevisiae and *G. glabrata* strains. We compared these data sets qualitatively with our sets by using Venn diagrams.

### GRACE library manipulations.

Selected strains from the GRACE library were tested for sensitivity or resistance to specific compounds. These strains were grown in the presence of 100.0 μg/ml tetracycline to inactivate the gene of interest and then tested for growth in the presence of the compound under study at the concentration used to treat the corresponding strains in the GRACE 1.0 collection.

## Supplementary Material

Supplemental material
