# Supplementary material for: Chemogenomic Profiling of the Fungal Pathogen Candida albicans
Source: Antimicrob Agents Chemother. 2018 Jan 25;62(2):e02365-17. doi: 10.1128/AAC.02365-17 (PMC5786791; doi:10.1128/AAC.02365-17)
Supplement: Supplemental material [file supp_62_2_e02365-17__index.html]

Supplemental material 

# Chemogenomic Profiling of the Fungal Pathogen Candida albicans

## Supplemental material

- Supplemental file 1 -

  Supplemental Tables S1, S3, S4, and S5 and Figures S1 and S2

  PDF, 7.5M
- Supplemental file 2 -

  Supplemental Table S2

  XLSX, 114K
- Supplemental file 3 -

  Supplemental Table S6

  XLSX, 75K
- Supplemental file 4 -

  Supplemental Table S7

  XLSX, 60K
