# Supplementary material for: Chemogenomic Profiling of the Fungal Pathogen Candida albicans
Source: Antimicrob Agents Chemother. 2018 Jan 25;62(2):e02365-17. doi: 10.1128/AAC.02365-17 (PMC5786791; doi:10.1128/AAC.02365-17)
Supplement: Supplemental material [file AAC.02365-17_zac002186900s1.pdf]

Table S1 : List of Oligonucleotides used in the manuscript

**Table S1**	
**Name**	**Sequence**
LR155F	5’-atttgAAAAATTCCAACAACTTAGGg-3’
LR155R	5’-aaaacCCTAAGTTGTTGGAATTTTTc-3’
LR156F	5’TGATTTGGgATCcGTTTTGAATGCACAACTGAGAAACCCCAAAAATTCCAACAACTaAtG-3’
LR156R	5’TAATCCATTATTATTATTATTACTAGTATTATTACTACttCaTtAGTTGTTGGAATTTTT-3’
LR157F	5’- GGAAGTCTTTGTAGATATAC-3’
LR157R	5’- CCATTTTTGAAGGATTTAGC-3’
LR135F	5’-CCATACACACACGTTAATTAG-3’
LR135R	5’-GCAGTTACTCTTTTTTTGGG-3’
LR182F	atttgGTGGCCCCTGAATTGTGTGCg*
LR182R	aaaacGCACACAATTCAGGGGCCACc*
LR183F	AGAAAGATGTTCCCGTGAAAAGTTCAACTACCGCTTGACGGTGGCCCCTtAATaGTGTGC*
LR183R	AGCATTGTATTTGTAGTCGTACTCTCTGACAAACAAGCttGCACACtATTaAGGGGCCAC*
LR184F	GTGTTGGAGAATAGACAGCG
LR184R	GCGGGTACATTCATTGTTGG
NPY1-S1	CCACCAAAAAAAAAATATTTTTCTCTTTCTATTTTAACATAGCTGCAATTTTTATTTCAACTTGTTCTTTTCTTTCTTTCTTTCTTTTGCTCTTCTTTGTGaagcttcgtacgctgcaggtc*
NPY1-exF	AAAACTATTGACTTAAACTC
NPY1-S2	CTAAATTTTTTTTTTTTGCAGCAAAAAGTTGAAAAAAGAAAAAGAACTCTTAAAACAACTCTAATGTATTTAATAATAATCTATCTCCTGTTCTACtctgatatcatcgatgaattcgag*
NPY1-exR	ATTTTAACAAACACTGATTT
X2-CaHIS1	CAACGAAATGGCCTCCCCTACCACAG
X3-CaHIS1	GGACGAA TTGAAGAAAGCTGGTGCAACCG
X2-CaARG4	AAT GGA TCA GTG GCA CCG GTG
X3-CaARG4	GAGGAGTACGACCTCAAGCGC

(**Table S2** in Excel format separately)

Table S3: In general the larger chromosomes (R, 1 and 2) show higher frequencies of LOH than the smaller chromosomes (5 and 6):

Table S3: Loss of heterozygosity (LOH) is co-related with the size of chromosomes, larger chromosomes showing higher frequencies of LOH.

	Chr1	Chr2	Chr3	Chr4	Chr5	Chr6	Chr7	ChrR
**Distribution of LOH**	3.40%	4.15%	2.50%	3.00%	1.30%	1.20%	2.50%	4.00%
**Chromosome Size**	3.2	2.2	1.8	1.6	1.2	1	0.95	2.5

Table S4: Lists of Genes common to the three species examined with different drug sensitivities:

Caspofungin Sensitivity:

**Genes common to *S.cerevisiae, C.albicans* and *C.glabrata***
MNT2

**Genes Common to *C.albicans* and *S.cerevisiae***	**Genes common to *S.cerevisiae* and *C.glabrata***	**Genes common to *C.albicans* and *C.glabrata***
SAC7	BCK1	CCH1
VPS24	ADA2	HEK2
BSD2	MKC1	SIP3
GOS1		
SWI4		
CAT20/STE20		

Fluconazole Sensitive:

**Genes common to *S.cerevisiae, C.albicans* and *C.glabrata***
None

**Genes Common to *C.albicans* and *S.cerevisiae***	**Genes common to *S.cerevisiae* and *C.glabrata***	**Genes common to *C.albicans* and *C.glabrata***
RPN4	None	None

Amphotericin B Sensitivity:

**Genes common to *S.cerevisiae, C.albicans* and *C.glabrata***
MNT2

**Genes Common to *C.albicans* and *S.cerevisiae***	**Genes common to *S.cerevisiae* and *C.glabrata***	**Genes common to *C.albicans* and *C.glabrata***
ATG15<br>RNH70<br>TPS1<br>MTG2<br>YUR1<br>RIT1<br>RBK1<br>POR1<br>ALG8<br>AYR1<br>HOG1<br>MNN10<br>LEN3	SSD1<br>DEP1<br>CWH41	None

Table S5: Lists of *C.albicans* strains used

Name of Strain	Genotype
CaSS1	his3::hisG/his3::hisG leu2::tetR-GAL4AD-URA3/LEU2
SN148	arg4Δ/arg4Δ, leu2Δ/leu2Δ, his1Δ/his1Δ, ura3Δ/ura3Δ
SN76	arg4Δ/arg4Δ, his1Δ/his1Δ, ura3Δ/ura3Δ

Table S6 and S7 are in Excel Format

Supplementary **Figure S1**:

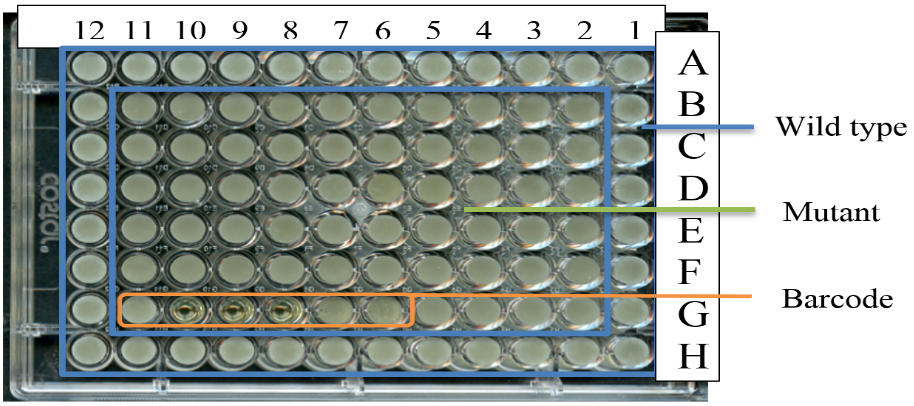

Fig. S1: A plate from Grace V1.0 collection showing the distribution of the strains in it. All the edges are occupied by the wild type parent strain CaSS1 (WT), and each plate has a unique barcode in the last 6 inner wells (G6 to G11) consisting of various patterns of CaSS1 and the morphologically distinct mutant *dig1/dig1*

Supplementary **Figure S2:**

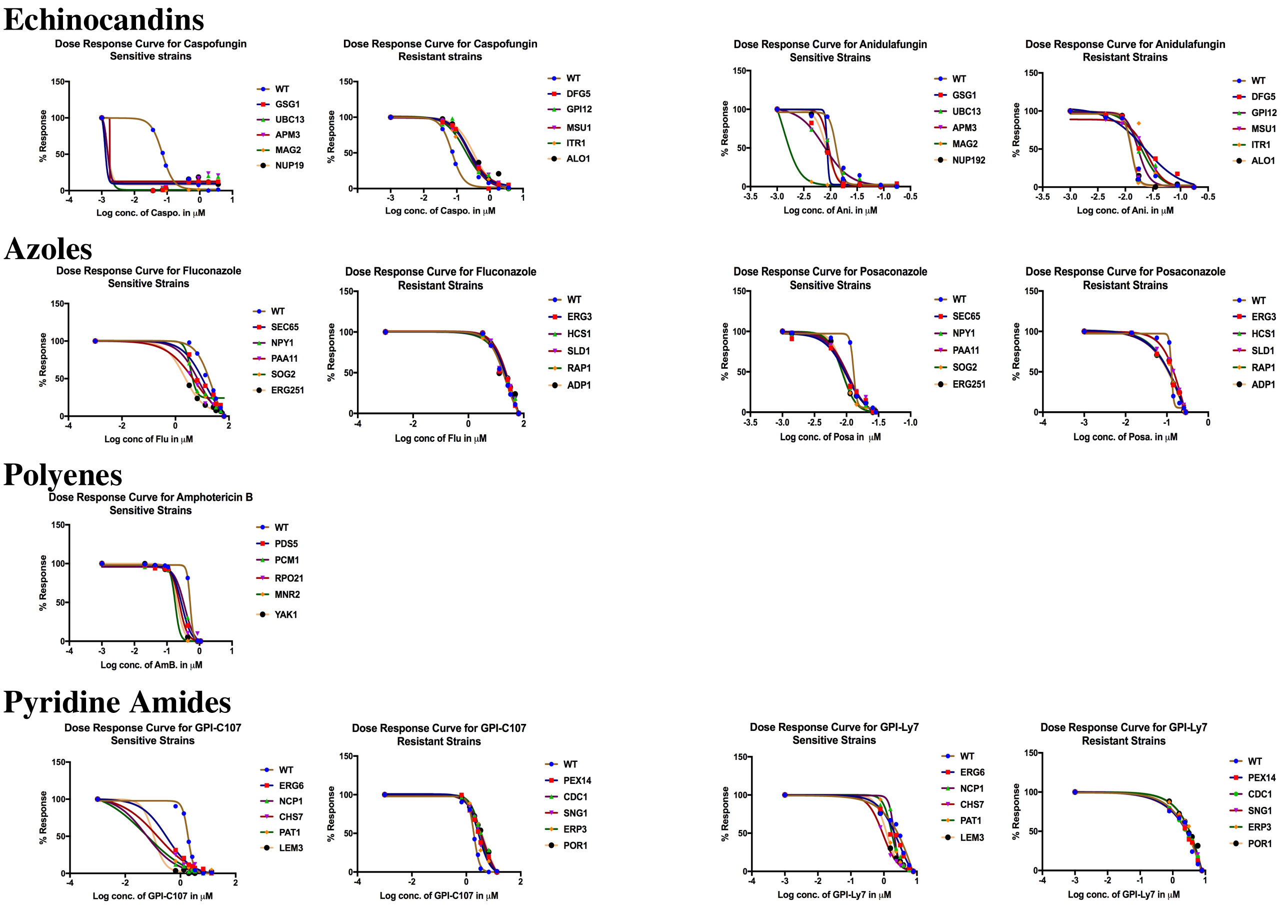

Fig S2: Dose response curves for the different drugs
